# Depressive symptoms and functional decline following coronary interventions in older patients with coronary artery disease: a prospective cohort study

**DOI:** 10.1186/s12888-016-0986-3

**Published:** 2016-08-04

**Authors:** M. Elizabeth Wilcox, Elizabeth A. Freiheit, Peter Faris, David B. Hogan, Scott B. Patten, Todd Anderson, William A. Ghali, Merril Knudtson, Andrew Demchuk, Colleen J. Maxwell

**Affiliations:** 1 Department of Medicine, Division of Respirology, University Health Network, Toronto, Canada; 2 Interdepartmental Division of Critical Care Medicine, University of Toronto, Toronto, Canada; 3 Institute of Social Research, University of Michigan, Ann Arbor, USA; 4 Department of Community Health Sciences, Cumming School of Medicine, University of Calgary, Calgary, Canada; 5 Research, Innovation and Analytics, Alberta Health Services, Foothills Medical Centre, Calgary, Canada; 6 Department of Medicine (Division of Geriatric Medicine), Cumming School of Medicine, University of Calgary, Calgary, Canada; 7 Department of Psychiatry and Mathison Centre for Mental Health Research and Education, Cumming School of Medicine, University of Calgary, Calgary, Canada; 8 Department of Medicine, Cumming School of Medicine, University of Calgary, Calgary, Canada; 9 Department of Cardiac Sciences, Cumming School of Medicine, University of Calgary, Calgary, Canada; 10 Libin Cardiovascular Institute of Alberta, University of Calgary, Calgary, Canada; 11 Departments of Clinical Neurosciences and Radiology, Hotchkiss Brain Institute, Cumming School of Medicine, University of Calgary, Calgary, Canada; 12 Schools of Pharmacy and Public Health and Health Systems, University of Waterloo, 200 University Avenue West, Waterloo, N2L 3G1 ON Canada; 13 Institute for Clinical Evaluative Sciences (ICES), Toronto, Canada

**Keywords:** Depression, Coronary artery disease, Coronary intervention, Functional outcomes, Functional decline, Cohort study

## Abstract

**Background:**

Depressive symptoms are prevalent in patients with coronary artery disease (CAD). It is unclear, however, how depressive symptoms change over time and the impact of these changes on long-term functional outcomes. We examined the association between different trajectories of depressive symptoms over 1 year and change in functional status over 30 months among patients undergoing coronary angiography.

**Methods:**

This was a prospective cohort study of 350 patients aged 60 and older undergoing non-emergent cardiac catheterization (October 2003–February 2007). A dynamic measure of significant depressive symptoms (i.e., Geriatric Depression Scale score 5+) capturing change over 12 months was derived that categorized patients into the following groups: (i) *no clinically important depressive symptoms* (at baseline, 6 and 12 months); (ii) *baseline-only symptoms* (at baseline but not at 6 and 12 months); (iii) *new onset symptoms* (not at baseline but present at either 6 or 12 months); and, (iv) *persistent symptoms* (at baseline *and* at either 6 or 12 month assessment). Primary outcomes were mean change in Older Americans Resources and Services (OARS) instrumental (IADL) and basic activities of daily living (BADL) scores (range 0–14 for each) across baseline (pre-procedure) and 6, 12, and 30 months post-procedure visits.

**Results:**

Estimates for the symptom categories were 71 % (none), 9 % (baseline only), 8 % (new onset) and 12 % (persistent). In adjusted models, patients with persistent symptoms showed a significant decrease in mean IADL and BADL scores from baseline to 6 months (−1.32 [95 % CI −1.78 to −0.86] and −0.63 [−0.97 to −0.30], respectively) and from 12 to 30 months (−0.79 [−1.27 to −0.31] and −1.00 [−1.35 to −0.65], respectively). New onset symptoms were associated with a significant decrease in mean IADL scores at 6 months and from 6 to 12 months. Patients with no depressive symptoms showed little change in scores whereas those with baseline only symptoms showed significant improvement in mean IADL at 6 months.

**Conclusions:**

Patients with persistent depressive symptoms were at greatest risk for worse functional status 30 months following coronary interventions. Proactive screening and follow-up for depression in this population offers prognostic value and may facilitate the implementation of targeted interventions.

**Electronic supplementary material:**

The online version of this article (doi:10.1186/s12888-016-0986-3) contains supplementary material, which is available to authorized users.

## Background

In older hospitalized adults, depressive symptoms are common and associated with a variety of adverse outcomes including worse quality of life [1, 2], reduced physical function [3], caregiver burnout [4], and increased mortality [5, 6]. Among older patients with coronary artery disease (CAD), estimates for minor and major depressive symptoms range from 30 to 45 % [7–9]. This compares to a prevalence of about 15 % among community-based samples of adults aged 60 years or older [10]. In CAD patients, depressive symptoms represent an independent risk factor for all-cause mortality and adverse cardiovascular events [7, 11, 12]. Although baseline measures of depression have been linked to poorer functional outcomes in older adults with CAD [13–15], most of this research has failed to consider the dynamic nature of depressive symptoms in this population or the associated consequences for longer-term health and functional outcomes. This is a significant knowledge gap as symptoms of depression may be transient, episodic or persistent [16]. With increasing survival rates among older adults with CAD undergoing coronary interventions [17], it is important to consider how the course of depressive symptoms may impact long-term functional independence in this vulnerable population.

In one of the few studies to explore this area, Sin and colleagues demonstrated a significant association between increases in depressive symptoms over 5 years and decreases in basic (but not instrumental) activities of daily living among 658 older adults with stable coronary heart disease [18]. In this study, changes in select disease severity markers (including frequency of angina and ejection fraction) were not found to be predictive of change in functional status. As this study was limited to older patients with stable disease and by an assessment of depression at baseline and at the 5-year follow-up only, further work to explore the trajectory and functional consequences of depressive symptoms in older patients undergoing coronary interventions is needed.

Emerging evidence suggests not all depressed patients with CAD are at risk of adverse health outcomes. Patients with new onset or persistent symptoms appear to be at highest risk for mortality and cardiac events [11, 19–21]. Among older community-dwelling adults, those exhibiting persistent depressive symptoms show an increased risk for cognitive and functional decline [22–24]. In a previous study of CAD patients undergoing coronary interventions, we observed that participants with persistent depressive symptoms had significantly greater declines at 30 months in attention/executive function, learning/memory, verbal fluency and global cognition [25]. Although others have examined predictors and outcomes of trajectories in depressive symptoms among patients with cardiovascular disease [2, 18], the impact of different trajectories in depressive symptoms assessed among CAD patients pre- and post-coronary interventions on functional change over a longer-term (beyond 1 year) remains to be explored. As we previously noted, while patients undergoing coronary interventions would be expected to exhibit significant variation in their symptoms, health status and well-being over time [25, 26], the impact of such variation on functional outcomes remain unknown.

Our aim was to examine the impact of change in clinically significant depressive symptoms over 1 year on long-term (30 month) change in instrumental (IADL) and basic activities of daily living (BADL) among older patients undergoing coronary catheterization who subsequently received coronary artery bypass graft (CABG) surgery, percutaneous coronary intervention (PCI) or medical therapy (MT).

## Methods

### Study Design

The Calgary Cardiac and Cognition (3C) Study was a prospective cohort investigation of the impact of neurocognitive and psychological factors on quality of life and functional status among older CAD patients undergoing evaluation for coronary revascularization.

Between October 2003 and May 2007 a total of 374 subjects aged 60 years and older were enrolled at a tertiary care hospital providing centralized cardiac services for residents of southern Alberta, Canada. Patients underwent coronary angiography (between October 2003 and February 2007) and post-catheterization, 128 underwent CABG surgery, 150 had PCI and 96 received MT. Trained cardiovascular research nurses screened all patients presenting for angiography for eligibility. Exclusion criteria included age < 60 years, emergency catheterization, prior revascularization and inability to provide informed written consent or complete the assessment due to language difficulties or severe cognitive and/or physical impairments. We purposefully oversampled those scheduled to undergo CABG and PCI (a comparison of our study sample to all eligible patients undergoing coronary catheterization during our recruitment period is available elsewhere) [25]. Ethics approval was obtained from the Conjoint Health Research Ethics Board, University of Calgary and informed consent was obtained from all participants.

A comprehensive standardized assessment including neuropsychological and physical performance tests, socio-demographic items, health behavior, self-rated health, activities of daily living and health-related quality of life measures was administered at baseline (pre-procedure), 6, 12, and 30 months post-procedure by trained research nurses or associates. Most (58 %) baseline assessments were conducted in hospital with the remainder as well as all follow-up assessments in the participant’s home. A trained psychometrician (blinded to patients’ clinical characteristics) reviewed and scored all cognitive testing. A structured interview with the patient’s identified informant (including Section H of the Cambridge Mental Disorders of the Elderly Examination-Revised [CAMDEX-R]) [27] was administered at all follow-up assessments, where possible. The study database was linked with the Alberta Provincial Project for Outcome Assessment in Coronary Heart Disease (APPROACH) [28], a comprehensive registry of all patients undergoing cardiac catheterization in the province, for baseline clinical characteristics and data on repeat revascularization(s) and mortality during follow-up. Three patients could not be linked because of out-of-province catheterizations (*n* = 2) or missing hospital records (*n* = 1). Specific details on the 3C assessment battery, variable coding and data collection procedures have been published elsewhere [25].

**Fig. 1:**
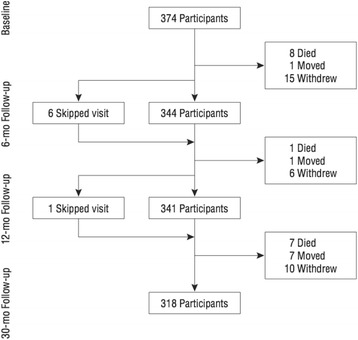
Calgary Cardiac and Cognition (3C) Study Flow. Legend/Footnote: Reproduced with permission from *Archives of General Psychiatry. 2012. 69(3):244–255*.^25^ Copyright © (2012) American Medical Association. All rights reserved

During the 30 months, 40 participants moved or withdrew, 16 died and 7 subjects missed either the 6 or 12 month assessment but remained in the study (Fig. 1). Loss to follow-up at 30 months was 11 % (40/374) of all enrolled subjects. The number of participants with outcome data at 6 and/ or 12 months and included in our analyses was 350/374 (94 %).

### Measurement of Depressive Symptoms

The 15-item Geriatric Depression Scale (GDS) [29, 30] with a cut-point of ≥ 5 was used to define clinically important depressive symptoms. The GDS is a commonly used screening test for depression in older adults with established validity and reliability [31–33]. It is well suited for medically ill older patients because of its focus on non-somatic symptoms. As done in previous studies [22, 25], we created a dynamic measure of significant depressive symptoms with the following categories: (i) *no clinically important depressive symptoms* (at baseline, 6 and 12 months); (ii) *baseline-only symptoms* (at baseline but not at 6 and 12 months); (iii) *new onset symptoms* (not at baseline but present at either 6 or 12 months); and, (iv) *persistent symptoms* (at baseline *and* at either the 6 or 12 month assessment).

### Functional Outcomes

Instrumental (IADL) and basic (BADL) activities of daily living were assessed with the Older Americans Resources and Services (OARS) Multidimensional Functional Assessment Questionnaire [34]. The IADL and BADL items demonstrate good reliability and validity and have been extensively utilized in past research on older adults [31]. The seven IADL items assess the respondent’s ability to use the telephone, travel distances independently, go shopping, prepare meals, do housework, take medicines and handle finances. The seven BADL items assess the respondent’s ability to feed self, dress/undress, groom self, walk, transfer to/from bed, bathe, and to use the bathroom. Response options were coded as 2 (can perform independently); 1 (requires some help/assistive device); or, 0 (is unable to perform on his/her own). Total scores for both scales ranged from 0 to 14, with lower scores indicating a higher level of impairment. IADL and BADL were examined as both continuous and binary (no vs. impairment [score of 0 or 1] in 1+ activities) variables.

### Other Measures

The socio-demographic, health and lifestyle characteristics of patients were assessed at baseline by study nurses [25]. Education was self-reported and recorded as number of completed full-time years since kindergarten. Anxiety was assessed with the State-Trait Anxiety Index [35] (state only), with higher scores indicating greater anxiety. Global cognitive performance was screened using the Mini-Mental State Exam (MMSE) [36]. Raw scores were used.

Baseline clinical data derived from the APPROACH database [28] included: admission diagnosis, ejection fraction, high risk coronary anatomy (i.e., double-vessel CAD with proximal left anterior descending artery involvement, any 3 vessel disease or left main disease), Canadian Cardiovascular Society (CCS) angina class, acute coronary syndrome, and disease history (cerebrovascular, congestive heart failure, peripheral vascular, diabetes mellitus, hypertension, hyperlipidemia, pulmonary, renal, malignancy, liver, gastrointestinal).

### Missing Data, Value Assignment and Imputation

Two participants (with dementia at follow-up) unable to complete the GDS were assigned GDS scores based on CAMDEX Part H [27] caregiver questions about the participant’s mood. Missing OARS data was rare with approximately 0.36 % missing across all visits for each of the individual IADLs and about 0.19 % missing for each of the individual BADLs. In each case, a geriatrician (DH) reviewed all data collected for the participant and assigned a score based on previous and subsequent OARS responses, current performance test results, cognitive test results, and quality of life responses.

### Statistical Analyses

Descriptive analyses were conducted to examine the distribution of patients’ socio-demographic and clinical characteristics overall and by depression status [25]. The 4-level categorical measure of depressive symptoms was examined with regard to mean change in continuous IADL and BADL scores between baseline and 6 months, 6 to 12 months, and 12 to 30 months using linear mixed models with a random intercept (function lmer in the lme4 package in R version 3.2.3) [37]. Restricted maximum likelihood (REML) was used to estimate the model parameters. To account for the impact of baseline covariates in changes over time, the models included interactions between time and age, sex, and baseline MMSE scores. We repeated these models also adjusting for a comorbidity measure (derived from selected diagnoses captured by the APPROACH database). As further adjustment for comorbidity did not alter our findings, we have presented the more parsimonious model estimates in this paper. The results were summarized in terms of generalized least squares (GLS) means with 95 % confidence intervals as well as mean differences with 95 % confidence intervals.

Secondary analyses used generalized estimating equations with an exchangeable correlation structure (function geeglm in the geepack package in R version 3.2.3) [37] to fit logistic regression models to evaluate changes in proportions of patients with any ADLs after accounting for baseline age, sex, and MMSE.

## Results

The mean age of our sample was 71.3 (SD 5.9) years and 73.7 % were male. Over 1 year, 248 (71 %) participants exhibited no significant depressive symptoms, 32 (9 %) had baseline-only symptoms, 28 (8 %) were new onset cases (at 6 or 12 months), and 42 (12 %) showed persistent symptoms. Table 1 presents a comparison of baseline characteristics (and follow-up GDS scores) across these four categories. There were relatively few meaningful differences between the groups. Compared to subjects without symptoms at any assessment: (i) those with new onset symptoms were older, less educated, and more likely to be living alone, have marked/unstable angina, or an acute coronary syndrome; and, (ii) those with persistent symptoms were more likely to report poor self-rated health, have higher anxiety scores, and report a history of diabetes, marked/unstable angina or an acute coronary syndrome. Baseline mean MMSE scores were significantly lower among all 3 depressive symptom groups compared to those with no symptoms.

**Table 1:** Baseline (and follow-up GDS) Characteristics of 3C Subjects by Depressive Symptom Change over 1 Year

	Depressive symptom category				
	None *n* = 248	Baseline only *n* = 32	New onset *n* = 28	Persistent *n* = 42	*P*-value*
Sociodemographic and Lifestyle Factors					
Age: mean (SD)	70.1 (5.8)	71.0 (6.1)	74.3 (6.5)	71.7 (5.3)	0.03
Age 75+: *n* (%)	57 (23.0)	7 (21.9)	9 (32.1)	12 (28.6)	0.11
Male: *n* (%)	186 (75.0)	23 (71.9)	18 (64.3)	31 (73.8)	0.67
Education: mean (SD), y	13.2 (3.8)	11.5 (2.7)	11.4 (4.3)	12.2 (4.2)	0.01
Lives alone: *n* (%)	31 (12.5)	8 (25.0)	9 (32.1)	7 (16.7)	0.02
Current or past smoker: *n* (%)	176 (71.0)	23 (71.9)	20 (71.4)	30 (71.4)	>0.99
Heavy drinker: *n* (%)	48 (19.4)	8 (25.0)	4 (14.3)	8 (19.0)	0.77
Treatment Group, *n* (%)					
CABG	80 (32.3)	13 (40.6)	10 (35.7)	18 (42.9)	0.66
PCI	107 (43.1)	13 (40.6)	9 (32.1)	14 (33.3)	
Medical Therapy	61 (24.6)	6 (18.8)	9 (32.1)	10 (23.8)	
Clinical Characteristics^a^ *n* (%)					
Admitted with stable angina (vs MI, unstable angina and other)	168 (68.3)	19 (61.3)	16 (57.1)	24 (57.1)	0.37
Ejection Fraction <50 %	53 (21.5)	9 (29.0)	6 (21.4)	9 (21.4)	0.82
High risk coronary anatomy^b^	105 (42.7)	19 (61.3)	16 (59.3)	20 (48.8)	0.17
CCS angina class > II	104 (42.3)	19 (61.3)	18 (64.3)	26 (61.9)	0.01
Acute coronary syndrome	53 (21.5)	9 (29.0)	11 (39.3)	16 (38.1)	0.04
Medical History^a^ *n* (%)					
Cerebrovascular disease	20 (8.1)	4 (12.9)	2 (7.1)	8 (19.0)	0.14
Congestive heart failure	22 (8.9)	3 (9.7)	5 (17.9)	3 (7.1)	0.45
Peripheral vascular disease^c^	21 (8.6)	2 (6.3)	1 (3.6)	7 (16.7)	0.57
Diabetes Mellitus (Type I or II)	52 (21.1)	11 (35.5)	4 (14.3)	15 (35.7)	0.05
Hypertension	185 (75.2)	27 (87.1)	23 (82.1)	33 (78.6)	0.44
Hyperlipidemia	207 (84.1)	24 (77.4)	25 (89.3)	34 (81.0)	0.62
Pulmonary disease	48 (19.5)	7 (22.6)	8 (28.6)	13 (31.0)	0.31
Renal disease	5 (2.0)	2 (6.5)	1 (3.6)	2 (4.8)	0.45
Malignancy	12 (4.9)	1 (3.2)	2 (7.1)	3 (7.1)	0.84
Severe/debilitating liver disease	1 (0.4)	1 (3.2)	0 (0)	0 (0)	0.23
Severe/debilitating gastrointestinal disease	14 (5.7)	3 (9.7)	2 (7.1)	7 (16.7)	0.09
Additional Clinical Information					
Baseline mean MMSE score (SD)	28.5 (1.3)	27.8 (2.0)	27.6 (2.3)	27.2 (2.6)	<0.001
Previous stroke and/or TIA: n(%)	27 (10.9)	4 (12.5)	5 (17.9)	7 (16.7)	0.57
Self rated health-fair/poor: n(%)^d^	34 (13.8)	16 (50.0)	7 (25.0)	23 (54.8)	<0.001
Anxiety level (STAI score): mean (SD)^e^	32.7 (9.7)	35.1 (9.2)	35.2 (10.2)	43.1 (10.2)	<0.001
Baseline and Follow-up GDS Score					
Baseline GDS scores, mean (SD)	1.76 (1.28)	6.22 (1.64)	2.39 (1.37)	7.60 (2.67)	<0.001
6 Month GDS scores, mean (SD)	1.12 (1.18)	2.35 (1.33)	5.61 (2.96)	7.19 (3.05)	<0.001
12 Month GDS scores, mean (SD)	1.18 (1.22)	1.78 (1.24)	4.26 (2.86)	6.90 (3.26)	<0.001
30 month GDS scores, mean (SD)	1.20 (1.46)	3.11 (2.25)	3.41 (2.89)	7.70 (3.70)	<0.001

Reproduced with permission from *Archives of General Psychiatry. 2012. 69(3):244–255*.^25^ Copyright © (2012) American Medical Association. All rights reserved

*Abbreviations*: *GDS* geriatric depression scale, *CABG* coronary artery bypass graft, *PCI* percutaneous coronary intervention, *MI* myocardial infarction, *MMSE* mini-mental state examination, *TIA* transient ischemic attack, *STAI*, State-Trait Anxiety Inventory

^a^APPROACH variables collected at time of catheterization, *n* = 246 patients in the group with no depressive symptoms and *n* = 31 in the group with symptoms at baseline only, otherwise noted

^b^ *n* = 27, *n* = 41 for the new onset and persistent groups respectively (high risk coronary anatomy)

^c^ *n* = 245 for the no depressive symptoms group (peripheral vascular disease)

^d^ *n* = 247 for no depressive symptoms group (self-rated health)

^e^ *n* = 246 for no depressive symptoms group (anxiety)

*F-test for continuous variables; chi-square test for categorical variables

Among participants with new onset and persistent depressive symptoms, there was an increase in the proportion with any IADL impairment from baseline to 30 months. There was little change among those with no depressive symptoms and a decrease in proportion with any IADL impairment among patients with baseline only symptoms (Table 2, for adjusted estimates see Additional file 1: Fig. S1). There was a higher proportion with any BADL impairment at 30 months evident among subjects with new onset and persistent symptoms (Table 2, for adjusted estimates see Additional file 1: Fig. S2).

**Table 2:** Percentage (95 % CI) of 3C Subjects with any IADL or BADL impairment at each assessment by Depressive Symptom Change over 1 Year

	Depressive symptom category			
	None	Baseline only	New onset	Persistent
IADL impairment				
Baseline^a^	16.9 (12.8–22.1)	53.1 (36.4–69.1)	28.6 (15.3–47.1)	35.7 (23.0–50.8)
6 Months^b^	21.0 (16.3–26.5)	25.8 (13.7–43.2)	50.0 (32.6–67.4)	50.0 (35.5–64.5)
12 Months^c^	16.2 (12.1–21.4)	25.0 (13.3–42.1)	44.4 (27.6–62.7)	54.8 (39.9–68.8)
30 months^d^	20.4 (15.6–26.1)	28.6 (15.3–47.1)	51.9 (34.0–69.3)	56.8 (40.9–71.3)
BADL impairment				
Baseline^a^	5.2 (3.1–8.8)	12.5 (5.0–28.1)	10.7 (3.7–27.2)	23.8 (13.5–38.5)
6 Months^b^	8.2 (5.4–12.4)	16.1 (7.1–32.6)	35.7 (20.7–54.2)	33.3 (21.0–48.4)
12 Months^c^	9.2 (6.1–13.5)	21.9 (11.0–38.8)	33.3 (18.6–52.2)	38.1 (25.0–53.2)
30 Months^d^	10.6 (7.2–15.3)	17.9 (7.9–35.6)	37.0 (21.5–55.8)	51.4 (35.9–66.6)

*Abbreviations*: *3C* calgary cardiac and cognition study, *IADL* instrumental activities of daily living, *BADL* basic activities of daily living

^a^sample sizes are *n* = 248, *n* = 32, *n* = 28, *n* = 42 for none, baseline only, new onset and persistent groups, respectively

^b^sample sizes are *n* = 243, *n* = 31, *n* = 28, *n* = 42 for none, baseline only, new onset and persistent groups, respectively

^c^sample sizes are *n* = 240, *n* = 32, *n* = 27, *n* = 42 for none, baseline only, new onset and persistent groups, respectively

^d^sample sizes are *n* = 226, *n* = 28, *n* = 27, *n* = 37 for none, baseline only, new onset and persistent groups, respectively

**Fig. 2:**
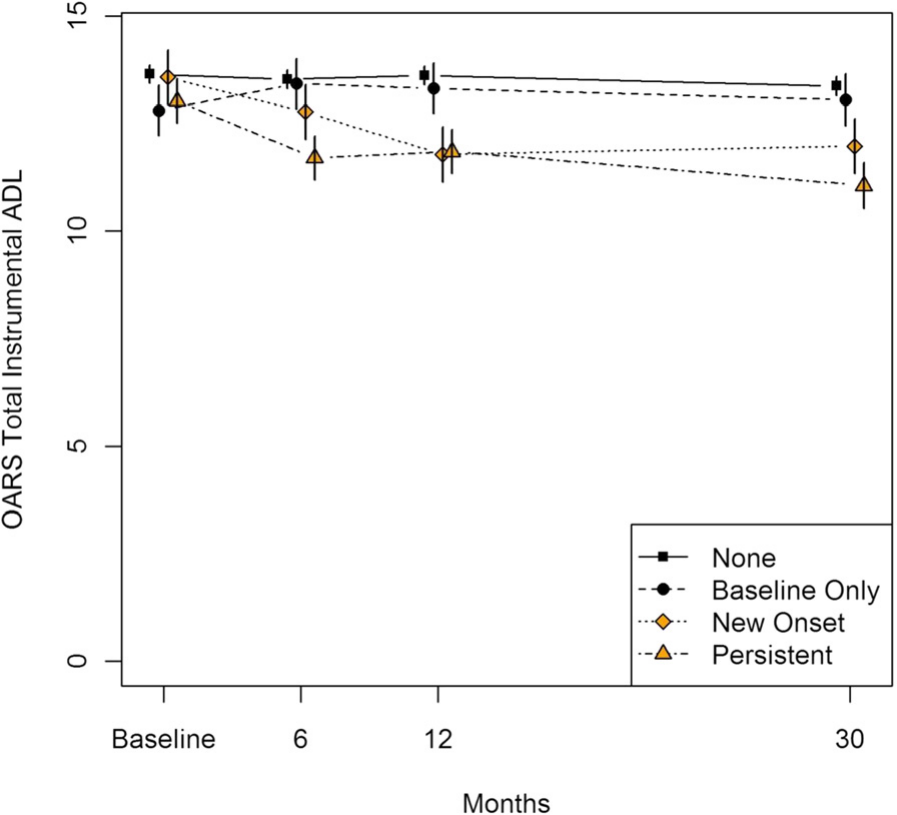
Generalized Least Squares Mean (95 % Confidence Interval) IADL^a^ Over Time, by Depressive Symptom Change over 1 Year. Legend/Footnote: ^a^adjusted for age, sex, and baseline MMSE score

**Fig. 3:**
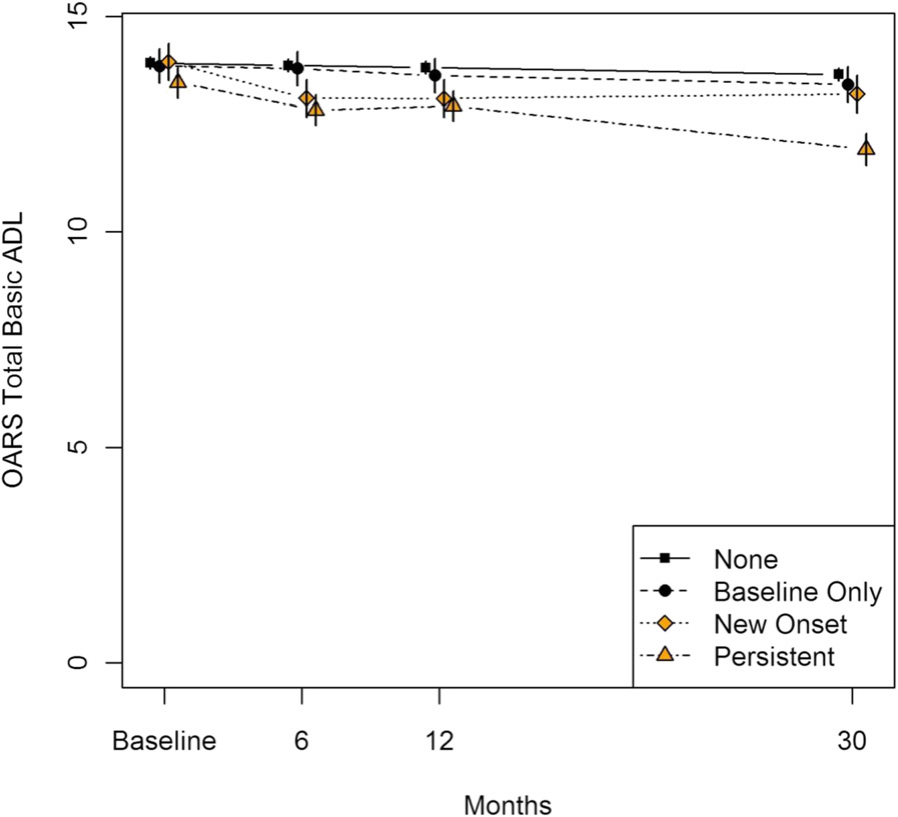
Generalized Least Squares Mean (95 % Confidence Interval) BADL^a^ Over Time, by Depressive Symptom Change over 1 Year. Legend/Footnote: ^a^adjusted for age, sex, and baseline MMSE score

In models adjusted for age, sex and baseline MMSE score, subjects with persistent depressive symptoms showed a significant decrease in mean IADL and BADL scores from baseline to 6 months (−1.32 [95%CI −1.78 to −0.86] and −0.63 [−0.97 to −0.30], respectively) and from 12 to 30 months (−0.79 [−1.27 to −0.31] and −1.00 [−1.35 to −0.65], respectively) post-procedure (Tables 3 and 4; Figs. 2 and 3). Those with new onset depressive symptoms showed a significant decrease in mean IADL and BADL scores from baseline to 6 months (−0.80 [−1.37 to −0.24] and −0.84 [−1.25 to −0.43], respectively) and in mean IADL score from 6 to 12 months (−0.99 [−1.56 to −0.42]) follow-up. Participants with baseline only depressive symptoms showed a significant improvement in mean IADL score from baseline to 6 months (0.62 [0.09 to 1.15]) and relative stability in mean IADL and BADL scores across subsequent follow-up assessments. Subjects exhibiting no depressive symptoms during the 1 year post-procedure showed no significant change in mean IADL or BADL scores from baseline to 6 months or from 6 to 12 months; however they exhibited a significant but small decrease in mean IADL and BADL scores from 12 to 30 months post-procedure (−0.24 [−0.44 to −0.05] and −0.16 [−0.30 to −0.02], respectively).

**Table 3:** Generalized Least Squares Mean (95 % Confidence Interval) Change in IADL^a^ Over Time, by Depressive Symptom Change over 1 Year

Depression symptom category	Mean		Change from previous measure		*P*-value
	Estimate	95 % CI	Estimate	95 % CI	
Baseline					
None	13.65	[13.44–13.86]	N/A	N/A	N/A
Baseline only	12.80	[12.22–13.39]	N/A	N/A	N/A
New onset	13.58	[12.94–14.21]	N/A	N/A	N/A
Persistent	13.03	[12.51–13.54]	N/A	N/A	N/A
6 months					
None	13.53	[13.32–13.74]	−0.12	[−0.31–0.07]	0.2118
Baseline only	13.43	[12.84–14.01]	0.62	[0.09–1.15]	0.0208
New onset	12.77	[12.14–13.41]	−0.80	[−1.37–−0.24]	0.0055
Persistent	11.71	[11.20–12.22]	−1.32	[−1.78–−0.86]	0.0000
12 months					
None	13.62	[13.41–13.83]	0.09	[−0.10–0.28]	0.3652
Baseline only	13.32	[12.74–13.90]	−0.11	[−0.63–0.42]	0.6904
New onset	11.79	[11.15–12.42]	−0.99	[−1.56–−0.42]	0.0007
Persistent	11.85	[11.34–12.36]	0.14	[−0.31–0.60]	0.5360
30 months					
None	13.37	[13.16–13.59]	−0.24	[−0.44–−0.05]	0.0146
Baseline only	13.06	[12.45–13.66]	−0.26	[−0.80–0.28]	0.3459
New onset	11.98	[11.34–12.61]	0.19	[−0.38–0.76]	0.5146
Persistent	11.06	[10.53–11.59]	−0.79	[−1.27–−0.31]	0.0013

^a^model adjusted for age, sex and baseline MMSE

**Table 4:** Generalized Least Squares Mean (95 % Confidence Interval) Change in BADL^a^ Over Time, by Depressive Symptom Change over 1 Year

Depression symptom category	Mean		Change from previous measure		*P*-value
	Estimate	95 % CI	Estimate	95 % CI	
Baseline					
None	13.92	[13.78–14.06]	N/A	N/A	N/A
Baseline only	13.85	[13.45–14.25]	N/A	N/A	N/A
New onset	13.94	[13.51–14.37]	N/A	N/A	N/A
Persistent	13.46	[13.11–13.81]	N/A	N/A	N/A
6 months					
None	13.86	[13.72–14.00]	−0.06	[−0.20–0.08]	0.4074
Baseline only	13.79	[13.39–14.19]	−0.06	[−0.44–0.32]	0.7538
New onset	13.10	[12.67–13.53]	−0.84	[−1.25–−0.43]	0.0001
Persistent	12.83	[12.48–13.17]	−0.63	[−0.97–−0.30]	0.0002
12 months					
None	13.81	[13.67–13.96]	−0.05	[−0.19–0.09]	0.4870
Baseline only	13.63	[13.23–14.02]	−0.16	[−0.54–0.22]	0.4068
New onset	13.09	[12.66–13.53]	−0.01	[−0.42–0.41]	0.9781
Persistent	12.92	[12.58–13.27]	0.09	[−0.24–0.43]	0.5738
30 months					
None	13.65	[13.50–13.80]	−0.16	[−0.30–−0.02]	0.0268
Baseline only	13.42	[13.01–13.83]	−0.21	[−0.60–0.18]	0.2942
New onset	13.20	[12.77–13.63]	0.10	[−0.31–0.52]	0.6213
Persistent	11.92	[11.55–12.28]	−1.00	[−1.35–−0.65]	0.0000

^a^model adjusted for age, sex and baseline MMSE

## Discussion

This study is one of but a few to explore the association between changes in depressive symptoms over time and long-term functional status among older CAD patients undergoing non-emergent catheterization. Patients with persistent symptoms experienced a significant decrease in mean IADL and BADL scores over the 30-month follow-up period post-procedure. A significant (but smaller) decrease in mean IADL and BADL scores during selected assessment times over follow-up was also evident among patients with new onset depressive symptoms. In contrast, there was relative stability in IADL and BADL functioning among patients with no depressive symptoms or depressive symptoms at baseline only. Among the latter group, there was a small but significant improvement in mean IADL scores between baseline and 6 months post-procedure.

The overall magnitude of change observed in our IADL and BADL measures during the 30 months appears to approximate a clinically meaningful difference in functional status [18, 38, 39]. In their recent study of depressive symptoms among older adults with stable coronary heart disease, Sin and colleagues [18] argued for the clinical relevance of a 1-point decrease in comparable IADL and BADL measures on the basis that a change of this magnitude would capture those needing assistance with an additional activity (or shifting to complete dependence from partial assistance for an activity).

Our findings are consistent with the hypothesis that persistent or more severe depressive symptoms are associated with worse outcomes in this patient population [11, 19–21]. Although there is extensive literature linking depression to disability among older community-based adults [24] and medical patients [40], a unique contribution of our work is the focus on the dynamic nature of depressive symptoms in a CAD population undergoing coronary interventions and subsequent risk of long-term functional outcomes. An earlier publication based on this cohort showed that participants with persistent depressive symptoms (relative to those with no or baseline-only symptoms) exhibited significantly greater decline at 30 months in multiple cognitive domains, including attention/executive functioning [25]. Executive dysfunction would be expected to place patients at heightened risk for greater functional disability [24, 41, 42]. In the current analyses, we show that persistent symptoms also represent an independent risk factor for long-term functional disability.

Several implications can be drawn from our work. First, depressive symptoms are not static among older adults with CAD undergoing coronary interventions. Some with clinically meaningful symptoms at baseline improve, while others have persistent problems that are associated with worse functional outcomes. The small but significant improvement in mean IADL scores observed between baseline and 6 months post-procedure among patients with depressive symptoms at baseline only illustrates this point. This sub-group may have experienced transient depressive symptoms in response to their health status pre-procedure or from anxiety about their diagnosis and impending procedure. The improvement in their disease symptoms and/or anxiety level post-procedure could explain both the absence of continued depressive symptoms and their increased likelihood for better functional status at 6 months. Our findings provide a rationale for assessing depressive symptoms at multiple time points (e.g., before and 6 or 12 months post-interventions). Information regarding new onset or persistent depressive symptoms could be used to delineate high-risk groups in need of targeted interventions to prevent or slow functional decline. The recognition of persistent symptoms may warrant greater secondary prevention efforts. Patients with persistent depressive symptoms may be at an increased risk for non-adherence [43] and consequently may require heightened surveillance. The significant improvement in IADL scores observed for patients with depressive symptoms at baseline only, suggests the potential for clinical interventions to alter functional trajectories.

Several possible mechanisms may explain why depressive symptoms are associated with worse functional status. Their presence could lead to functional limitations. Experienced depression may decrease motivation for, and reward from, physical and/or social activities important for the maintenance of functional independence. This is consistent with the finding that depressed patients are more difficult to engage in physical therapy, often crucial for recovery of function [44]. Depressive symptoms can amplify symptoms of medical illnesses [45], or affect adherence to medication regimens [43], worsening the course of various chronic conditions. Further, depressive symptoms might affect functioning through neuroendocrine and inflammatory mechanisms [46]. It is possible that depression may negatively influence a patient’s perceptions of what they are able to do [47], limiting their ability to participate in rehabilitation programs. Conversely, impaired function might also lead to depressive symptoms because of the perceived loss of independence and mastery. We suspect the relationship between the two is bi-directional. Finally, both depressive symptoms and functional impairments might be secondary to factors such as small vessel cerebrovascular disease [48], worse control of the patients CAD [49, 50], or the presence (and impact) of other co-morbidities [51].

Strengths of our study include the longitudinal design and minimal lost to follow-up despite a long duration (30-month period), availability of detailed and repeat measures of patients’ clinical, cognitive and functional status, and inclusion of all three treatment options (CABG, PCI and MT) following catheterization in the 3C cohort. Our interpretations are limited by the observational nature of the study and some uncertainty about the clinical significance of the differences seen in IADL and BADL scores. The relatively small number of cases for a number of the categories limited our ability to include numerous covariates in our adjusted analyses. We were unable to explore sex and/or ethnic differences in our analyses because most patients were male and 95 % were of European or unknown ethnicity. Due to the absence of information, we were also not able to explore the influence of a previous history of depressive symptoms, use of selected therapies (e.g., antidepressants, psychotherapy, cardiac rehabilitation) or the availability of social support on the observed associations between the evolution of depressive symptoms and functional decline. At the same time, it is noteworthy that there were few meaningful differences in the sociodemographic or clinical characteristics between patients with baseline only compared with persistent depressive symptoms, despite clear differences in their functional outcomes. As well, adjustment for variation in comorbidity (in addition to age, sex and MMSE scores) did not alter our findings. We believe the parsimonious approach we took to modeling was appropriate in this study as further adjustment for other covariates that were not observed to vary by depressive symptom category or that might be considered possible mediators of the association between depressive symptom change and functional decline may have posed a risk of unnecessary adjustment (leading to a loss of precision in estimates) or overadjustment bias (leading to a masking of relevant exposure—outcome associations), respectively [52]. Finally, our findings may have limited generalizability to other patient populations including CAD patient populations not undergoing invasive procedures, as all patients underwent coronary catheterization.

## Conclusions

In our sample of older CAD patients undergoing coronary interventions, significant decreases in mean functional scores over the 30 month follow-up were observed for those with persistent (but not baseline only) depressive symptoms. This suggests a one-time assessment of depressive symptoms may be inadequate for determining which patients are at high-risk for adverse functional outcomes. Although there is little available research regarding the impact of depression treatment on functional outcomes, our findings illustrate the need for longer-term monitoring and further large-scale evaluation of management strategies (pharmaceutical and non-pharmaceutical) for depression in CAD patients. A number of recent studies have shown that a collaborative care model, where both depression and cardiovascular disease are simultaneously managed in the primary care setting with the aid of a consulting psychiatrist, may result in a significant (albeit modest) reduction in depressive symptoms [53, 54]. Whether or not such multifaceted interventions have a meaningful impact on the quality of life and functional outcomes of older CAD patients remains to be determined.

### Main Bullet Points

- Patients with persistent depressive symptoms were at greatest risk for worse functional status (assessed as decline in instrumental [IADL] and basic [BADL] activities of daily living) 30 months following coronary interventions.
- Patients with no depressive symptoms or depressive symptoms at baseline only showed relative stability in their IADL and BADL functioning over time.
- Repeated screening for depression in older patients with coronary artery disease offers prognostic value and may facilitate the implementation of targeted strategies to optimize long-term functional outcomes.

## Abbreviations

3C, calgary cardiac and cognition study; APPROACH, Alberta Provincial Project for Outcome Assessment in Coronary Heart Disease; BADL, basic activities of daily living; CABG, coronary artery bypass graft; CAD, coronary artery disease; CAMDEX-R, Cambridge Mental Disorders of the Elderly Examination-Revised; CCS, Canadian Cardiovascular Society; GDS, geriatric depression scale; GLS, generalized least squares; IADL, instrumental activities of daily living; MMSE, mini-mental state exam; MT, medical therapy; OARS, Older Americans Resources and Services; PCI, percutaneous coronary intervention; REML, restricted maximum likelihood; SD, standard deviation; STAI, State-Trait Anxiety Index

## Additional file

Additional file 1: Figure S1. (Percentage [95 % confidence interval] of 3C Subjects with any IADL impairment at each assessment by depressive symptom change over 1 year); **Figure S2.** (Percentage [95 % confidence interval] of 3C Subjects with any BADL impairment at each assessment by depressive symptom change over 1 year). (DOCX 297 kb)
