# Supplementary material for: Depressive symptoms and functional decline following coronary interventions in older patients with coronary artery disease: a prospective cohort study
Source: BMC Psychiatry. 2016 Aug 4;16:277. doi: 10.1186/s12888-016-0986-3 (PMC4973530; doi:10.1186/s12888-016-0986-3)
Supplement: Additional file 1: Figure S1. — (Percentage [95 % confidence interval] of 3C Subjects with any IADL impairment at each assessment by depressive symptom change over 1 year); Figure S2. (Percentage [95 % confidence interval] of 3C Subjects with any BADL impairment at each assessment by depressive symptom change over 1 year). (DOCX 297 kb) [file 12888_2016_986_MOESM1_ESM.docx]

**Figure S1.** Percentage (95% CI) of 3C Subjects with any IADL impairment^a^ at each assessment

by Depressive Symptom Change over 1 Year.


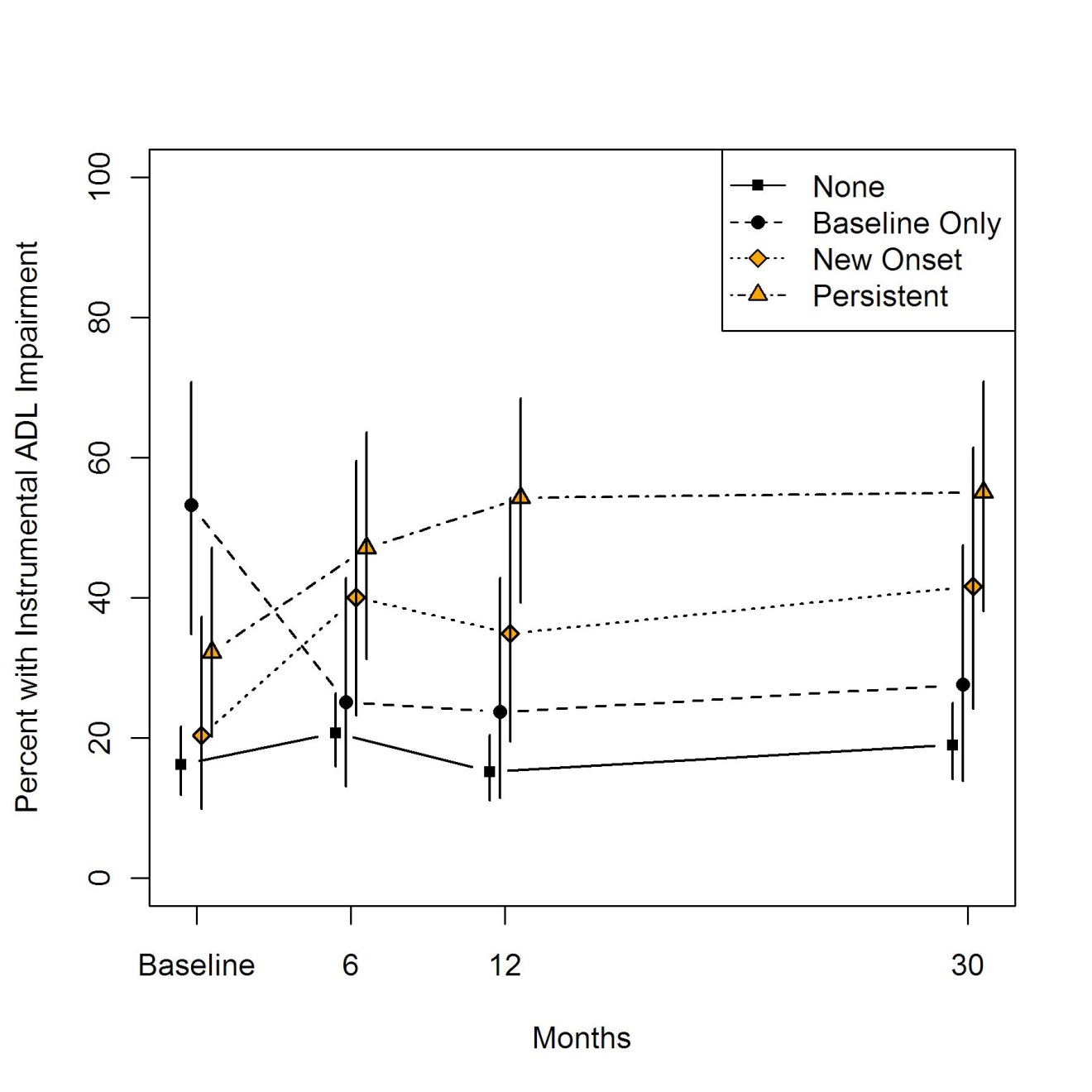


^a^adjusted for age, sex and baseline MMSE

|  | **Baseline** | **6** | **12** | **30** |
| --- | --- | --- | --- | --- |
| None | 16.21 | 20.69 | 15.20 | 19.00 |
| Baseline Only | 53.21 | 25.14 | 23.70 | 27.63 |
| New Onset | 20.37 | 40.05 | 34.91 | 41.61 |
| Persistent | 32.23 | 47.12 | 54.24 | 55.02 |
| Adjusted Percentage of Patients with any IADL over time by Depression Category | | | | |

**Figure S2.** Percentage (95% CI) of 3C Subjects with any BADL impairment^a^ at each assessment

by Depressive Symptom Change over 1 Year.


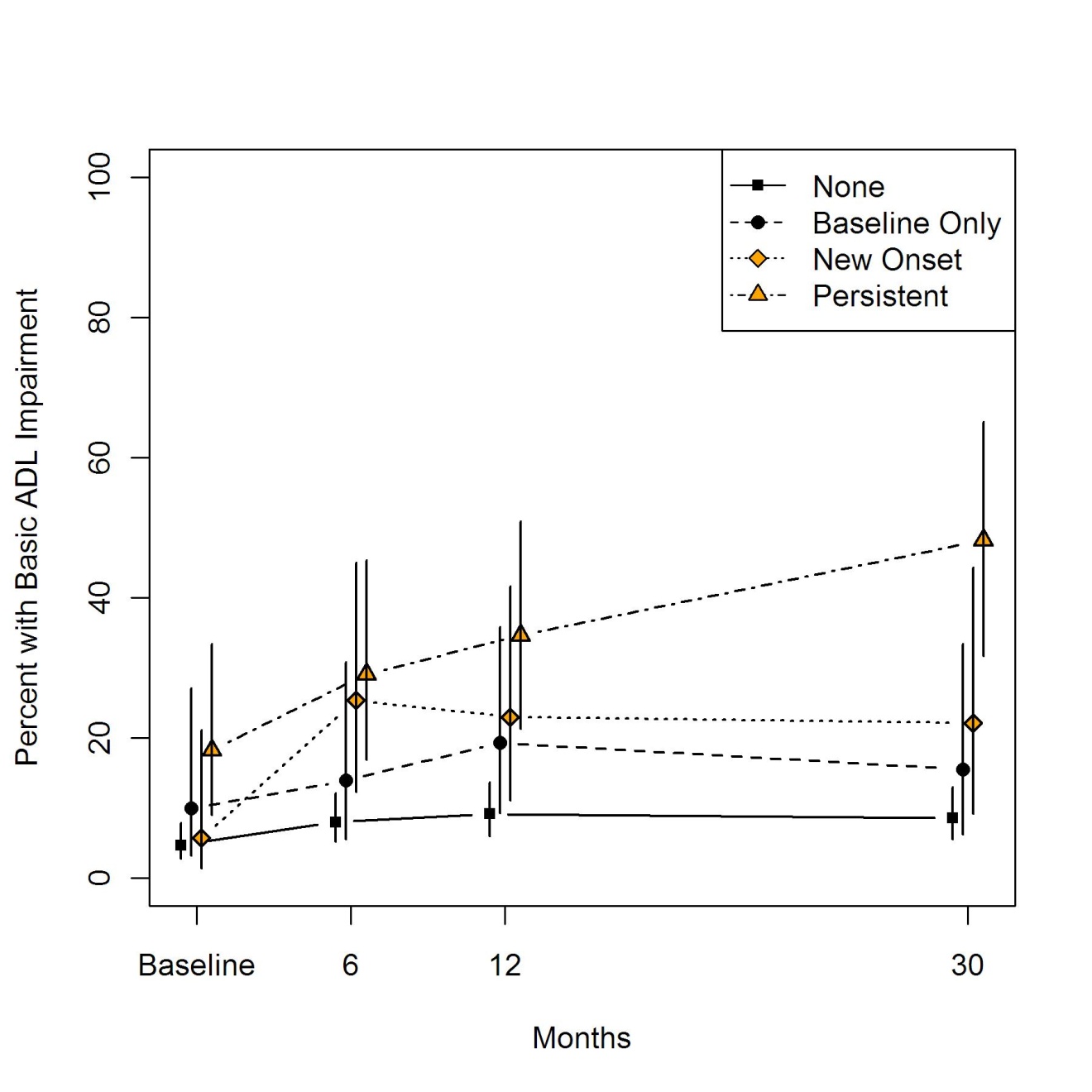


^a^adjusted for age, sex and baseline MMSE

|  | **Baseline** | **6** | **12** | **30** |
| --- | --- | --- | --- | --- |
| None | 4.70 | 8.02 | 9.16 | 8.55 |
| Baseline Only | 9.97 | 13.92 | 19.33 | 15.50 |
| New Onset | 5.76 | 25.35 | 22.99 | 22.13 |
| Persistent | 18.24 | 29.10 | 34.64 | 48.23 |
| Adjusted Percentage of Patients with any BADL over time by Depression Category | | | | |
